# Photocatalytic Degradation and Antibacterial Properties of Fe^3+^-Doped Alkalized Carbon Nitride

**DOI:** 10.3390/nano10091751

**Published:** 2020-09-04

**Authors:** Ying Gao, Jizhou Duan, Xiaofan Zhai, Fang Guan, Xiutong Wang, Jie Zhang, Baorong Hou

**Affiliations:** 1 Key Laboratory of Marine Environmental Corrosion and Bio-Fouling, Institute of Oceanology, Chinese Academy of Sciences, Qingdao 266071, China; yinggaogy@163.com (Y.G.); guanfang@qdio.ac.cn (F.G.); wangxiutong@qdio.ac.cn (X.W.); zhangjie@qdio.ac.cn (J.Z.); brhou@qdio.ac.cn (B.H.); 2 University of Chinese Academy of Sciences, Beijing 100049, China; 3 Open Studio for Marine Corrosion and Protection, Pilot National Laboratory for Marine Science and Technology (Qingdao), Qingdao 266071, China; 4 Center for Ocean Mega-Science, Chinese Academy of Sciences, Qingdao 266071, China

**Keywords:** g-C_3_N_4_, alkalization, Fe^3+^-doping, Rhodamine B degradation, antibacterial property

## Abstract

Discovering novel materials and improving the properties of existing materials are the main goals in the field of photocatalysis to increase the potential application of the materials. In this paper, a modified graphitic carbon nitride (g-C_3_N_4_) photocatalyst named Fe^3+^-doped alkalized carbon nitride, which couples the photocatalytic reaction with the Fenton reaction, is introduced to demonstrate its Rhodamine B (RhB) degradation and antibacterial properties. Under visible-light irradiation, the degradation rate of RhB was 99.9% after 200 min, while the antibacterial rates of *Pseudomonas aeruginosa* (*P. aeruginosa*), *Escherichia coli* (*E. coli*), and *Staphylococcus aureus* (*S. aureus*) after 300 min were 99.9986%, 99.9974%, and 99.9876%, respectively. Moreover, the repetitive experiments of RhB degradation demonstrate that the proposed photocatalysts have excellent stability and reusability. The active free radical trapping experiments reveal that the superoxide radical ($\cdot O_{2}^{-}$) is the dominant reactive oxygen species. In addition, the Fenton reaction is introduced into the photocatalytic system due to the doping of Fe^3+^, and the hydroxyl radical (·OH) produced from the Fenton reaction further enhances the photocatalytic performance. The remarkable improvement in photocatalytic performance of the proposed photocatalyst can be attributed to its broader UV–visible absorption characteristic and the occurrence of the Fenton reaction.

## 1. Introduction

Photocatalytic materials have great potential applications for decomposing organic contaminants, killing bacteria in the environment and preventing marine biofouling [1,2]. Therefore, photocatalytic technology, with energy-saving features, is an effective strategy to solve environmental challenges. However, traditional photocatalysts cannot reach high photocatalytic efficiencies due to their narrow visible light response range [3,4,5]. Therefore, developing novel photocatalysts with high catalytic efficiencies in visible light is an important issue [6,7]. Graphitic carbon nitride (g-C_3_N_4_), which is a stable metal-free photocatalyst with a bandgap of 2.7 eV and capability for light absorption up to 460 nm, is an appropriate candidate for solving environmental problems [8,9]. Nonetheless, the photocatalytic efficiency of pure g-C_3_N_4_ is limited because of the high recombination rate of photoinduced carriers [10]. To enhance the visible light catalytic efficiency of g-C_3_N_4_, several strategies—nanostructured g-C_3_N_4_ synthesis [11,12], metal deposition [13,14], nonmetal doping [15,16,17], protonation [18], and compounding with other materials [19,20,21,22]—have been applied to modify g-C_3_N_4_. These methods can effectively enhance the separation efficiency of the photoinduced carrier and the light response range of g-C_3_N_4_. If other reactions that can generate active free radicals are introduced into the catalytic system on this basis, then the catalytic rate of the catalytic system will be higher.

The Fenton process, an advanced oxidation process, is widely applied in industrial wastewater treatments due to the abundant ·OH generated by the redox reactions between H_2_O_2_ and Fe species [23]. In addition, the Fenton reaction has been extensively investigated for the degradation of organic pollutants [24]. However, both H_2_O_2_ and Fe species must exist simultaneously for the occurrence of the Fenton reaction. In this regard, although semiconductor photocatalysis could be utilized to catalyze the production of H_2_O_2_ by two-electron reduction of O_2_, the H_2_O_2_ generation rate is very low (<0.1 mM·h^−^^1^) [25]. This occurs because the semiconductor mainly promotes the one-electron reduction of O_2_ to form superoxide radical (·OOH) (Equation (1)) or the four-electron reduction of O_2_ to form H_2_O (Equation (2)), while the two-electron reduction of O_2_ to form H_2_O_2_ (Equation (3)) is suppressed [26].

(1) $$O_{2}+H^{+}+e^{-}\to \cdot \mathrm{OOH}\;\;-0.13\;V\;\mathrm{vs}.\;\mathrm{NHE}$$

(2) $$O_{2}+4H^{+}+4e^{-}\to 2H_{2}O\;\;1.23\;V\;\mathrm{vs}.\;\mathrm{NHE}$$

(3) $$O_{2}+2H^{+}+2e^{-}\to H_{2}O_{2}\;0.68\;V\;\mathrm{vs}.\;\mathrm{NHE}$$

where NHE is the normal hydrogen electrode potential. g-C_3_N_4_ could promote the two-electron reduction of O_2_ to form H_2_O_2_ under visible light irradiation owing to its suitable bandgap and band edge position, but pure g-C_3_N_4_ has very low activity to produce H_2_O_2_ [27,28,29]. Compared with pure carbon nitride (CN), alkalized carbon nitride (AKCN) has a wider visible light response range. Moreover, it has been proven that photogenerated carriers in AKCN can efficiently be separated with directional charge migration and that AKCN can selectively reduce O_2_ to H_2_O_2_ due to its almost 100% apparent quantum yield [29]. Therefore, doping Fe species in AKCN rather than directly in g-C_3_N_4_, when Fe species are doped in AKCN, the Fenton reaction is introduced into the photocatalytic system to enhance the photocatalytic property of AKCN. In addition, there are abundant “nitrogen pots” consisting of six nitrogen atoms in g-C_3_N_4_-based materials that can be used as active trapping sites for encapsulating metal ions, such as doping Fe species into AKCN [30]. For example, Li et al. doped Fe species into AKCN by an impregnation method, which coupled photocatalysis with the Fenton reaction [31]. However, since the Fe^3+^ was decorated on the shallow surface of AKCN, the Fe^3+^ was easily removed after repeating the experiment several times. Therefore, it is important to discover a facile method for introducing Fe species into AKCN to form a stable structure during its synthesis procedure.

In this paper, pure g-C_3_N_4_ (CN), alkalized carbon nitride (AKCN) and Fe^3+^-doped alkalized carbon nitride (AKCN-xFe) were synthesized, and the catalytic activities of the prepared semiconductor photocatalysts were investigated in terms of Rhodamine B (RhB) degradation and bacterial sterilization under visible light irradiation. Furthermore, AKCN-xFe coupled the photocatalytic reaction with the Fenton reaction. Moreover, the photocatalytic mechanism of AKCN-xFe was proposed by radical scavenger experiments and energy band calculations.

## 2. Experimental

### 2.1. Materials

Melamine, potassium hydroxide (KOH), potassium chloride (KCl), ferric chloride (FeCl_3_), dibasic sodium phosphate (Na_2_HPO_4_), potassium dihydrogen phosphate (KH_2_PO_4_), sodium hydroxide (NaOH), sodium sulfate (Na_2_SO_4_), sodium chloride (NaCl), peptone, yeast extract and agar powder were purchased from Sinopharm Chemical Reagent Co. Ltd. (Shanghai, China). Rhodamine B (RhB), 2-propanol (IPA), tert-butyl alcohol (TBA), sodium oxalate (MSDS), 1,4-benzoquinone (BQ) and terephthalic acid (TA) were purchased from Shanghai Macklin Biochemical Co. Ltd. (Shanghai, China). All chemicals were of analytical grade and were not further purified.

### 2.2. Preparation of the Photocatalysts

#### 2.2.1. Synthesis of Carbon Nitride (CN)

Melamine (6 g) was calcinated at 550 °C for 4 h with a heating rate of 2.5 °C·min^−1^ in a muffle furnace. After heating treatment, the resulting product was ground, filtered, washed, and dried at 60 °C.

#### 2.2.2. Synthesis of Alkalized Carbon Nitride (AKCN)

The synthesis process was the same as that of CN. However, KOH (2.0 mmol) and KCl (0.08 mol) were blended with melamine (1 mol) and ground together before calcination. This molar ratio was selected based on the results reported by Zhang et al. [29] to obtain AKCN with the highest photocatalytic performance.

#### 2.2.3. Synthesis of Fe^3+^-Doped Alkalized Carbon Nitride (AKCN-xFe)

Here, x represents the molar weight of doped Fe^3+^. Melamine (1 mol), KOH (2.0 mmol), KCl (0.08 mol), and different amounts of FeCl_3_ (0.006, 0.010, and 0.014 mol) were mixed and ground together before calcination. The obtained Fe^3+^-doped alkalized carbon nitrides were labeled AKCN-0.006Fe, AKCN-0.010Fe and AKCN-0.014Fe.

### 2.3. Characterization

The morphologies of the catalysts were considered by scanning electron microscopy (SEM; ULTRA 55, Zeiss, Oberkochen, Germany). The nanosheet thickness was measured by atomic force microscope (AFM; afm5500, Agilent, Palo Alto, CA, USA). The microstructures of the catalysts were observed by transmission electron microscopy (TEM; JEM-2010F, JEOL, Tokyo, Japan). The X-ray diffraction pattens of the catalysts were detected by an X-ray diffractometer (XRD; max-3C, Rigaku D, Tokyo, Japan) operating under Cu-Kα radiation. The scanned 2θ ranged from 10° to 60° with a scanning speed of 5°·min^−1^. X-ray photoelectron spectra (XPS) were recorded with XPS equipment (Escalab250Xi, Thermo Fisher, Waltham, MA, USA). The infrared transmission spectra were obtained using a Fourier Transform infrared (FTIR) spectrophotometer (Nicolet iS10, Thermo, Waltham, MA, USA) assisted with KBr pellets. The ultraviolet–visible absorption spectra were measured on a UV–visible spectrophotometer (U-4100, Hitachi, Tokyo, Japan), and barium sulfate (BaSO_4_) was taken as the reference. The photoluminescence spectrum of 2-hydroxyterephthalic acid (TA-·OH) at an excitation wavelength of 365 nm was obtained by a fluorescence spectrometer (F-4600, Hitachi, Tokyo, Japan). An electrochemical workstation (CHI660E, Chenhua, Shanghai, China) was employed to measure the Mott–Schottky plots, photocurrent, and electrochemical impedance spectroscopy (EIS) of the as-prepared catalysts. Electrochemical measurements were conducted by a conventional three-electrode system. Indium tin oxide (ITO) glass was used to prepare the working electrode. Sample powder (3 mg) and ethanol (1 mL) were ultrasonically dispersed, and the resultant slurry was then drop-coated on the ITO plate with a fixed area of 10 mm × 10 mm. After drying at 120 °C for 30 min, the strong adhesion of the photocatalyst to the ITO substrate was noticeable. The electrolyte was distilled water containing Na_2_SO_4_ (0.1 M). The working electrode, Pt counter electrode, and Ag/AgCl reference electrode were then immersed in the solution. To minimize the effect of the material layer thickness, the working electrode was irradiated from its backside (ITO glass/semiconductor interface). For the Mott–Schottky measurement, the disturbance signal was set to 10 mV [32]. A bias voltage of 0.5 V was set to drive the photogenerated electrons. EIS was measured at open circuit potential with frequency range of 10^5^–10^−2^ Hz.

### 2.4. Photocatalytic Experiments

The photocatalytic degradation performance of the as-prepared photocatalysts was investigated by degradation of Rhodamine B (RhB) solution. A 500 W Xenon lamp with a cutoff filter (λ > 420 nm) was selected as the light source. In the experiment, 50 mg photocatalyst was dispersed in a quartz tube containing 50 mL of RhB solution (10 mg·L^−1^). Before irradiation, the solution was stirred in the dark for 30 min to reach adsorption–desorption equilibrium. Then, 4 mL of the solution was periodically collected, and the remnant concentration of RhB was determined by analyzing the absorption peak at 554 nm using UV–visible absorption spectroscopy. The photocatalytic degradation rate (%) of RhB was calculated by means of Equation (4).

(4) $${\mathrm{DR}}_{\mathrm{RhB}}=\left(1-C_{t}/C_{0}\right)\times 100$$

where $C_{0}$ is the original concentration of RhB and $C_{t}$ is the concentration at reaction time t [33].

The photocatalytic antibacterial performance was measured against the model bacteria *Pseudomonas aeruginosa* (*P. aeruginosa*)*, Escherichia coli* (*E. coli*), and *Staphylococcus aureus* (*S. aureus*) [34]. *P. aeruginosa, E.coli* and *S. aureus* were cultured on solid Luria–Bertani (LB) medium containing NaCl (10 g·L^−^^1^), peptone (10 g·L^−^^1^), yeast extract (5 g·L^−^^1^) and agar powder (20 g·L^−^^1^) for 24 h at 37 °C. The bacterial colony formed on the solid medium was first inoculated in liquid LB medium (10 g·L^−1^ NaCl, 10 g·L^−1^ peptones, and 5 g·L^−1^ yeast extract) and then cultured at 37 °C for 12 h. After culture, the bacteria are separated from the liquid LB medium by centrifugation (6000 rpm·min^−1^ for 5 min) and washed twice with phosphate-buffer saline (PBS; 8.0 g·L^−1^ NaCl, 0.2 g·L^−1^ KCl, 1.44 g·L^−1^ Na_2_HPO_4_, 0.44 g·L^−1^ KH_2_PO_4_ in distilled water). The concentrations of *P. aeruginosa*, *E. coli* and *S. aureus* in the liquid LB medium were 10^8^ cfu·mL^−1^, which were determined by counting colonies. The light source used in this experiment was the same as the light source used in the photodegradation experiment. Typically, photocatalyst (50 mg), PBS (49.5 mL), and bacterial suspension (500 µL) were added into quartz tubes in sequence. The solution was stirred in the dark for 30 min before irradiation. Then, 1.0 mL of the solution was collected every 60 min and diluted with PBS. To count the number of bacterial colonies, 100 µL of this solution is cultured on the solid LB plates for 24 h at 37 °C. The survival rate was calculated by Equation (5).

(5) $$\mathrm{survival}\;\mathrm{rate}\;\left(\%\right)=\left(N_{t}/N_{0}\right)\times 100$$

where N_0_ and N_t_ are the numbers of bacteria in the blank control test without photocatalysts and in the samples with photocatalysts, respectively. Therefore, the antibacterial rate can be defined by Equation (6).

(6) antibacterial rate (%)=100−survival rate

All media were sterilized at 121 °C for 30 min. An AIRTECH clean bench was applied after UV light irradiation for 30 min during the inoculation and assembly system.

Active free radical trapping experiments: 2-propanol (IPA) [35] and tert-butyl alcohol (TBA) [36,37] were the scavengers of hydroxyl radical (·OH), sodium oxalate (MSDS) [38] and 1,4-benzoquinone (BQ) [39] were used as hole (h^+^) and superoxide radical ($\cdot O_{2}^{-}$) scavengers. The experimental procedure was the same as the photocatalytic activity experiments with the addition of 1 mmol scavenger [39]. The concentration of ·OH generated from the Fenton reaction was estimated by detecting fluorescent TA-·OH at 435 nm [40]. The as-prepared photocatalyst was dispersed in 50 mL of terephthalic acid (TA)/NaOH solution ($5\times 10^{-4}$ mol·L^−1^, $2\times 10^{-3}$ mol·L^−1^) under visible light irradiation [24]. 

## 3. Results and Discussion

### 3.1. Morphology

**Figure 1:**
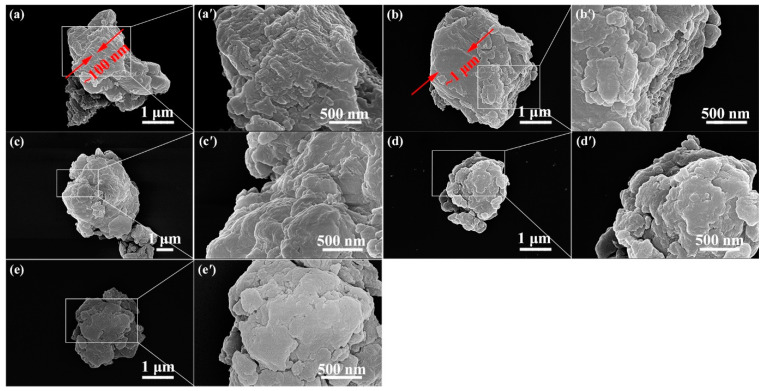
SEM images of (**a**) pure g-C_3_N_4_ CN, (**b**) alkalized carbon nitride (ACNK), (**c**) Fe^3+^-doped alkalized carbon nitride (AKCN-0.006Fe), (**d**) AKCN-0.010Fe, and (**e**) AKCN-0.014Fe ((**a**′–**e**′) are the corresponding local enlargements).

Figure 1 shows the SEM images of the synthesized samples. All the samples possess nanosheet-like morphology, indicating the successful formation of the graphitic stacking structure. In comparison to CN (Figure 1a′), AKCN and AKCN-xFe have clearer and looser lamellar morphologies (Figure 1b′–e′), implying that the interspacing between the layers has increased after alkalization and Fe^3+^ doping. Moreover, as shown by the red arrows in Figure 1a,b, the area of a single nanosheet of AKCN is larger than that of CN, which can be attributed to the increased malleability of nanosheets after carbon nitride alkalization. Although the nanosheet area decreases after Fe^3+^ iron deposition (Figure 1c–e), the clear layered morphology remains unchanged (Figure 1c′–e′), suggesting that the Fe^3+^ modification has some effects on the exfoliation and loosening of nanosheets and increases the specific surface area [41]. A larger specific surface could promote light harvesting and the generation of charge carriers; a larger specific surface area could also accelerate electrons transport and provide more reactive sites [42]. The sample thickness was measured by AFM, and the corresponding results are presented in Figure S1. The thickness of CN and AKCN were approximately 10 nm (Figure S1a,b). The thickness decreased after Fe^3+^ doping, and the thicknesses of AKCN-0.006Fe, AKCN-0.010Fe, and AKCN-0.014Fe were 7 nm, 3 nm, and 2 nm, respectively (Figure S1c–e). The reduction of the material thickness also indicates that Fe^3+^ doping caused the exfoliation and loosening of the nanosheets (Figure S2).

### 3.2. Structure and Composition

The elemental compositions of different photocatalysts determined by EDS are presented in Figure S3, confirming the successful alkalization and Fe^3+^ doping. Fe amounts of 0.29%, 0.34%, and 0.86% were estimated for AKCN-0.006Fe, AKCN-0.010Fe and AKCN-0.014Fe, respectively (Table S1).

**Figure 2:**
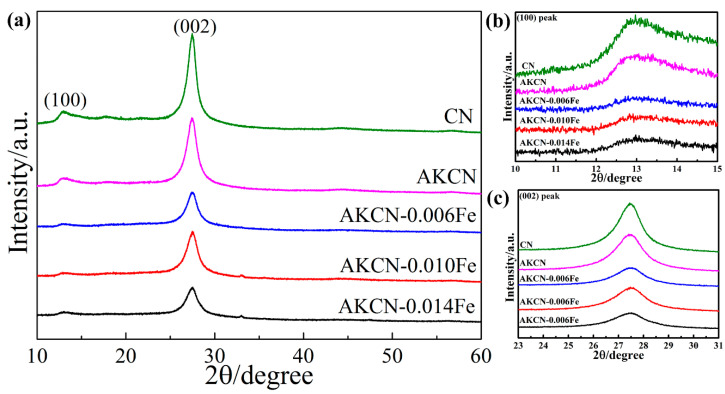
(**a**) XRD patterns of the synthesized semiconductor photocatalysts, and enlarged view of (**b**) (100) peak and (**c**) (002) peak.

The XRD patterns of the synthesized photocatalysts represented in Figure 2 described the effects of alkalinization and Fe^3+^ deposition on the structure of g-C_3_N_4_. The XRD pattern of CN displays two typical peaks at around 13.0° and 27.4°. The XRD patterns of AKCN and AKCN-xFe are similar to that of CN (Figure 2a). The main (002) and minor (100) peaks originated from in-plane repeated tri-s-triazine units and interlayer stacking of conjugated aromatic CN units, respectively [43]. After alkalization and Fe^3+^ doping, the (002) peak experiences a slight downshift (Figure 2c), suggesting that the distance between every two layers has increased and the conjugated aromatic system is loose. In addition, the observed decrease in the intensity of the (002) peak signifies that the spacing between g-C_3_N_4_ layers has increased [44]. It is possible that the interaction of ions in different layers has changed the interspace between layers. The weak (100) signal at 13.0° in Figure 2b indicates that K^+^ ions are embedded into the in-plane and interlayers of CN sheets, resulting in a strong interaction between K and N [45,46]. A significant decrease in the intensity of (002) and (100) peak is observed after Fe^3+^ doping, indicating that Fe^3+^ ions exist in the in-planes and interlayers of CN sheets. In addition, the weakening of the (100) peak after Fe^3+^ doping confirms the strong interaction between Fe atoms and tri-s-triazine groups [13].

**Figure 3:**
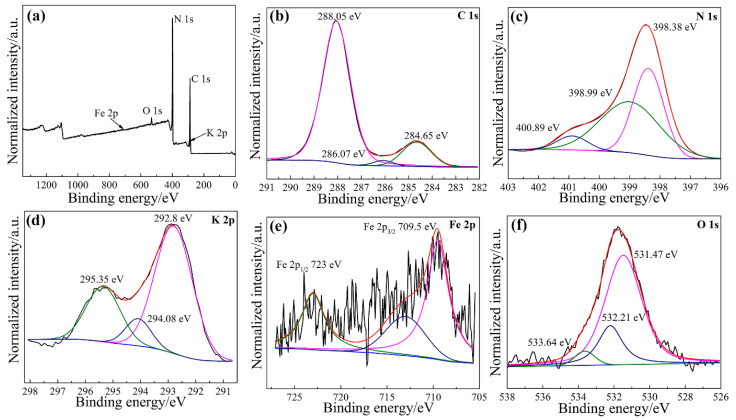
X-ray photoelectron spectra (XPS) spectra of AKCN-0.010Fe: (**a**) survey, (**b**) C 1s, (**c**) N 1s, (**d**) K 2p, (**e**) Fe 2p, and (**f**) O 1s.

XPS analysis was conducted to study the chemical state in CN, AKCN and AKCN-xFe. Figure 3a reveals that AKCN-0.010Fe contains C, N, K, Fe, and O. Due to the very low Fe^3+^ content in AKCN-0.010Fe, the peaks of Fe species are not detected. Figure 3b shows that the C 1s spectrum is divided into three components. The peaks at 284.65 eV, 286.07 eV, and 288.05 eV correspond to the sp^2^ C-C bonds, C-NH_x_, and sp^2^-bond carbon ((N-)_2_C=N), respectively [47,48,49]. The N 1s spectrum in Figure 3c also consists of three parts. The peaks at 398.38 eV, 398.99 eV, and 400.89 eV can be identified as sp^2^ hybridized N atoms (edge nitrogen atoms) in the C=N-C bonds of triazine rings, sp^3^ hybridized N atoms (inner and bridge nitrogen atoms) in the N-(C)_3_ group, and amino NH_x_ (NH_2_ or NH) groups [50,51]. Figure S4 shows that most peaks of N 1s and C 1s in AKCN have shifted to lower binding energies in comparison to those of CN, and this phenomenon reveals that the modification of KCl and KOH has affected the chemical structure of CN [29]. In addition, Fe^3+^ ions changed the chemical structure of AKCN because most N 1s and C 1s peaks in AKCN-0.010Fe shifted to lower binding energies in those samples than in AKCN (Figure S4). Figure 3d displays the K 2p spectrum. The peak at 292.8 eV can be ascribed to N-K bonds that link K to N atoms in different layers [31], which is consistent with the XRD result that K^+^ ions are embedded into the in-plane and interlayers of CN sheets. The Fe 2p spectrum is illustrated in Figure 3e. The chemical state of the Fe species can be identified as trivalent because the binding energy of Fe 2p_3/2_ (709.5 eV) is approximately equal to the reported value for Fe^3+^ [31]. A small O 1s peak in the survey scan appeared from the graft of oxygen-containing species (-C-OH) on the surface of materials [29]. In Figure 3f, the O 1s peak at 533.64 eV corresponds to C-OH and indicates that hydroxyl groups are grafted on AKCN-xFe [52].

**Figure 4:**
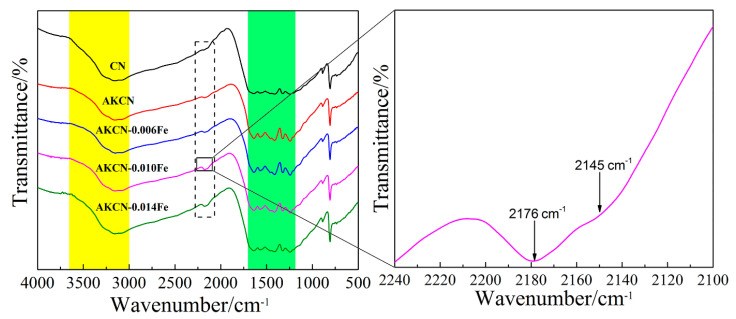
FTIR spectra of the synthesized semiconductor photocatalysts.

FTIR analysis was performed to verify the grafting of hydroxyl groups. All samples show the typical patterns of g-C_3_N_4_ (Figure 4). The absorption bands in the wavelength ranges of 1200–1700 cm^−1^ and 3000–3700 cm^−1^ correspond to the typical stretching modes of CN heterocycles and the stretching vibration modes of adsorbed H_2_O or residual N-H. The peak at 806 cm^−1^ and 885 cm^−1^ are assigned to the representative breathing mode of the s-triazine ring system and the deformation mode of N-H [53]. Two extra bands at 2176 cm^−^^1^ and 2145 cm^−^^1^ reveal the steady grafting of hydroxyl groups, and the signal of these bands is stronger in AKCN because the terminal -NH_2_ groups were replaced by hydroxyl groups after the introduction of KCl and KOH [54]. Furthermore, the signals of two extra bands in AKCN-xFe are stronger than those in AKCN, implying that Fe^3+^ doping facilitates the grafting of hydroxyl groups. This behavior can be assigned to the exfoliation resulting from Fe^3+^ doping as well as increasing the specific surface area of AKCN-xFe, leading to easier grafting of hydroxyl groups on AKCN-xFe.

### 3.3. Band Structure

**Figure 5:**
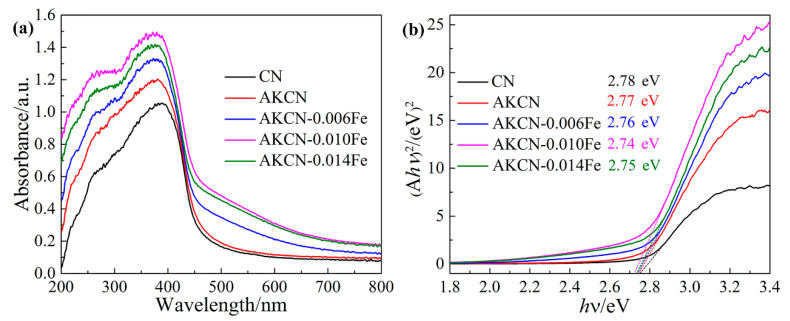
(**a**) UV–visible absorption spectra and (**b**) bandgaps of the synthesized semiconductor photocatalysts.

Optical characterization was utilized to depict the band structures of the proposed g-C_3_N_4_-based materials. The UV–visible absorption spectra of the as-prepared photocatalysts are shown in Figure 5a. The absorption edge of AKCN-xFe has redshifted in comparison to those of CN and ACNK, indicating that the visible light response range is wider after Fe^3+^ doping. Indeed, the decrease in bandgap (Eg) generally could cause a redshift in light absorption [55]. The Eg values of the as-prepared photocatalysts are calculated by the Tauc plot method with index r = 2 (Figure 5b) [56,57]. The calculated Eg values for CN, ACNK, AKCN-0.006Fe, AKCN-0.010Fe, and AKCN-0.014Fe are 2.78 eV, 2.77 eV, 2.76 eV, 2.74 eV, and 2.75 eV, respectively, which are the typical recorded bandgaps for g-C_3_N_4_-based materials. The results reveal that the bandgap width has decreased after alkalization and Fe^3+^ doping, improving the catalytic efficiency of g-C_3_N_4_-based materials; i.e., when the bandgap is reduced, less energy is required for transition of photogenerated electrons from valence band (VB) to conduction band (CB) [58].

**Figure 6:**
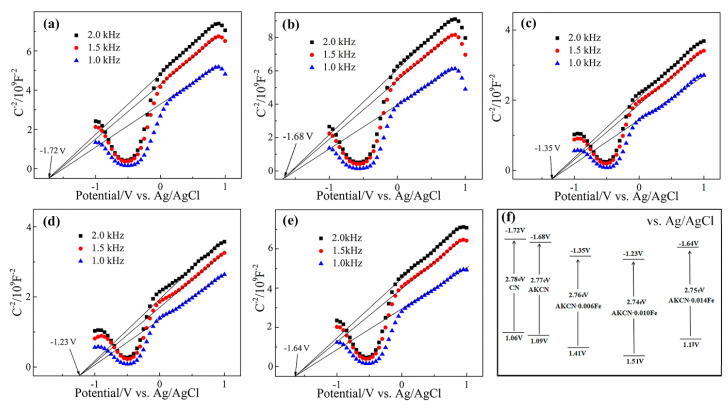
Electrochemical Mott–Schottky plots of (**a**) CN, (**b**) AKCN, (**c**) AKCN-0.006Fe, (**d**) AKCN-0.010Fe, and (**e**) AKCN-0.014Fe; (**f**) electronic band structures of different samples.

The semiconductor type and flat band potential of the synthesized photocatalysts were determined by analyzing the Mott–Schottky plots. The selected frequencies are 1 kHz, 1.5 kHz, and 2 kHz, and the Mott–Schottky plots of the samples are exhibited in Figure 6a–e. The positive slope of the Mott–Schottky curves indicates that all the synthesized samples are n-type semiconductors. The CB and VB potentials of the as-prepared photocatalysts were obtained according to the curves of the bandgap width as well as the Mott–Schottky plots (Figure 6f). It is obvious that the VB shifted positively after alkalization and Fe^3+^ doping, and the VB of AKCN-0.010Fe was the most positive. The positive shift of the VB after doping may be caused by the replacement of C with doped atom. When the dosage of FeCl_3_ was 0.014 mol, the bandgap width of AKCN-0.014Fe was wider than that of AKCN-0.010Fe, and their CB shifts negatively compared to that of AKCN-0.010Fe. Since doping can produce stress, the excessive stress caused by excessive Fe^3+^ doping leads to the negative shift of the CB.

### 3.4. Photodegradation Performance

**Figure 7:**
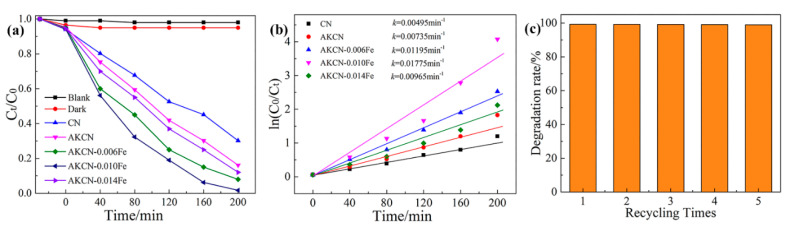
(**a**) Degradation efficiencies of the as-prepared semiconductor photocatalysts toward Rhodamine B (RhB) (**b**) dynamics of degradation reactions, and (**c**) recycling experiments of RhB photocatalytic degradation.

The photodegradation performance of the synthesized semiconductor photocatalysts under visible light irradiation was considered by using RhB as the model organic pollutant. According to Figure 7a, there was no obvious degradation of RhB for either the blank experiment (i.e., the sample was exposed to visible light conditions without a photocatalyst) as well as dark experiment (i.e., the sample was under dark conditions with a photocatalyst), revealing that the influence of light and other natural factors on pollutant degradation can be ignored. Figure 7a shows that the AKCN-xFe can more efficiently degrade RhB than CN and AKCN due to its broader UV–visible absorption properties. Furthermore, approximately 99.9% of RhB was degraded by AKCN-0.010Fe under visible light irradiation for 200 min, which shows a higher efficiency than AKCN-0.006Fe (91.9%) and AKCN-0.014Fe (88.1%). When Fe^3+^ was introduced within the structure, the separation of photogenerated carriers was promoted because the empty electron orbit of Fe^3+^ captures the photoelectrons of the matrix. We can find from the photocurrent curves of the prepared photocatalysts in Figure S5a that the carrier separation rate of AKCN-xFe is faster, especially AKCN-0.010Fe. The smallest EIS semicircular of AKCN-0.010Fe in Figure S5b indicates that its interface charge transfer is the fastest [59]. Moreover, the doping of Fe^3+^ would suppress the light absorption capability of the semiconductor host. Therefore, the best photodegradation performance of AKCN-xFe can be achieved by adjusting the Fe^3+^ content to an optimal value. However, when the Fe^3+^ doping concentration was higher than the optimal value, the electrons and holes were captured at the same time, and the doped ions could easily reunite. This phenomenon will increase the recombination chance of carriers and decrease the photocatalytic activity. Accordingly, considerable attention must be paid to the amount of doped Fe^3+^ and the dispersion of Fe^3+^ ions in semiconductor materials. In addition, g-C_3_N_4_-based photocatalytic materials can catalyze the production of H_2_O_2_; hence, the Fenton reaction occurs in the presence of Fe^3+^ ions and produces more ·OH, which has the highest oxidation activity. AKCN-0.010Fe satisfies the conditions where the Fenton reaction occurs and contains optimal Fe^3+^ content; hence, it manifests the highest catalytic activity in RhB degradation among all the synthesized samples. Kinetic analysis cannot be neglected in the photocatalytic degradation of any organic compound, and an important reaction rate constant can be obtained. In the present study, kinetic analysis was performed to reveal the photodegradation of RhB under the function of g-C_3_N_4_-based catalysts. All photocatalytic degradation reactions followed the pseudo-first-order kinetics (Equation (7)) [60].

(7) $$\ln\left(C_{0}/C_{t}\right)=kt$$

where *k* is the first-order rate constant, C_0_ is the original concentration of RhB, C_t_ is the concentration of RhB at reaction time *t*, and *k* is calculated by linear regression of the slope of the $\ln\left(C_{0}/C_{t}\right)$ vs. time curve Figure 7b [61,62,63]. The reaction rate constant of AKCN-0.010Fe (*k* = 0.01775 min^−1^) was higher than those of CN (*k* = 0.00495 min^−1^), AKCN (*k* = 0.00735 min^−1^), AKCN-0.006Fe (*k* = 0.01195 min^−1^), and AKCN-0.014Fe (*k* = 0.00965 min^−1^), which intuitively shows that AKCN-0.010Fe had a faster catalytic reaction rate. The reason for the higher reaction rate in the abovementioned sample is that the empty electron orbit of Fe^3+^ captures photogenerated electrons, which accelerates the separation of photogenerated carriers and improves the visible light catalytic performance of AKCN-0.010Fe. Stability and reusability are the main criteria for photocatalysts. The stability and reusability of AKCN-0.010Fe were evaluated by repetitive experiments of RhB degredation. During the repetitive experiments, AKCN-0.010Fe was separated by centrifuge after each experiment and then washed and dried. It is noticeable from Figure 7c that after five cycles, AKCN-0.010Fe exhibited no significant decrease in the degradation rate of RhB.

### 3.5. Photocatalytic Antibacterial Performance

**Figure 8:**
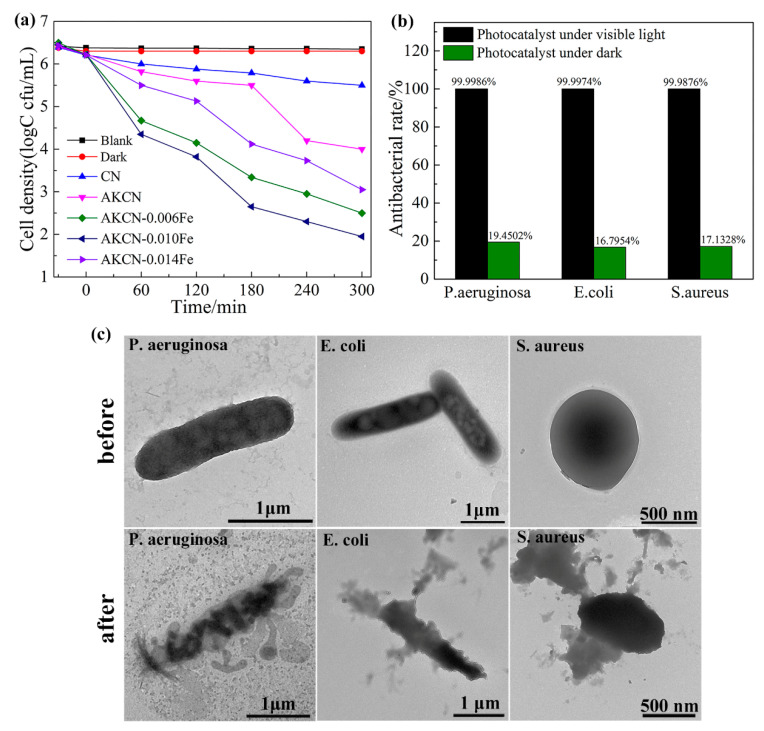
(**a**) Survival curves of *P. aeruginosa* as a function of the as-prepared semiconductor photocatalysts under visible light and (**b**) photocatalytic antibacterial rate histogram towards *P. aeruginosa, E. coli*, and *S. aureus* under visible light. (**c**) TEM images of the *P. aeruginosa, E. coli*, and *S. aureus* cell before and after photocatalytic reaction.

**Figure 9:**
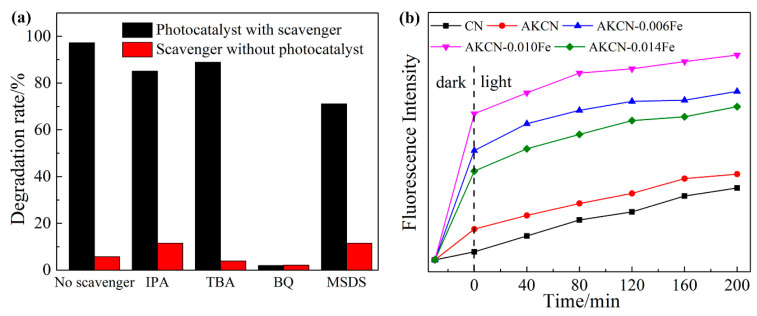
(**a**) Radical scavenger experiments for the degradation of RhB with AKCN-0.010Fe under visible light and (**b**) hydroxyl radicals trapping fluorescence (PL) spectra in terephthalic acid solution under visible light.

In the present experiment, the fouling bacterium *P. aeruginosa* (5.3 × 10^6^ cfu·mL^−1^) was used to detect the photocatalytic antibacterial properties of CN, AKCN, and AKCN-xFe under visible light irradiation. The survival curve of *P. aeruginosa* in Figure 8a reveals the quantity of bacteria after irradiation. The number of bacteria basically remains constant in the blank and dark experiments, indicating that the proposed photocatalysts are nontoxic and the effects of visible light on bacteria can be ignored. AKCN-0.010Fe exhibits the best antibacterial efficiency in comparison to pure CN, AKCN, AKCN-0.006Fe, and AKCN-0.014Fe under visible light. It is noticeable from Figure 8a that AKCN-0.010Fe killed almost all the bacteria, while the antimicrobial rate of *P. aeruginosa* reached 99.9986% after 300 min of irradiation. The optimal photocatalytic performance of AKCN-0.010Fe can be attributed to its higher separation efficiency of photogenerated carriers. In addition, the widest range of visible light absorption of AKCN-0.010Fe has a significant contribution to its optimum photocatalytic capability. AKCN-0.010Fe generates the highest amount of ·OH through the Fenton reaction (Figure 9b), which also enhances the photocatalytic performance. Furthermore, Gram-negative *E. coli* ($2.3\times 10^{6}$ cfu·mL^−1^) and Gram-positive *S. aureus* ($3.7\times 10^{6}$ cfu·mL^−1^) were selected to verify the broad-spectrum antibacterial properties of AKCN-0.010Fe. It is noticeable from Figure 8b that the antimicrobial rates of AKCN-0.010Fe towards *E. coli* and *S. aureus* reached 99.9974% and 99.9876%, respectively, after 300 min of visible light irradiation. Furthermore, the appearance of bacteria was observed by TEM, and the results indicated that the cell walls and membranes of normal *P. aeruginosa*, *E. coli* and *S. aureus* were smooth and that their cytoplasm was distributed evenly before the experiment. However, after the experiment, the cell wall and membranes of these bacteria were severely damaged, and the cytoplasm outflowed, indicating that the bacteria were killed during the experiment (Figure 8c).

### 3.6. Photocatalytic Mechanism

**Scheme 1:**
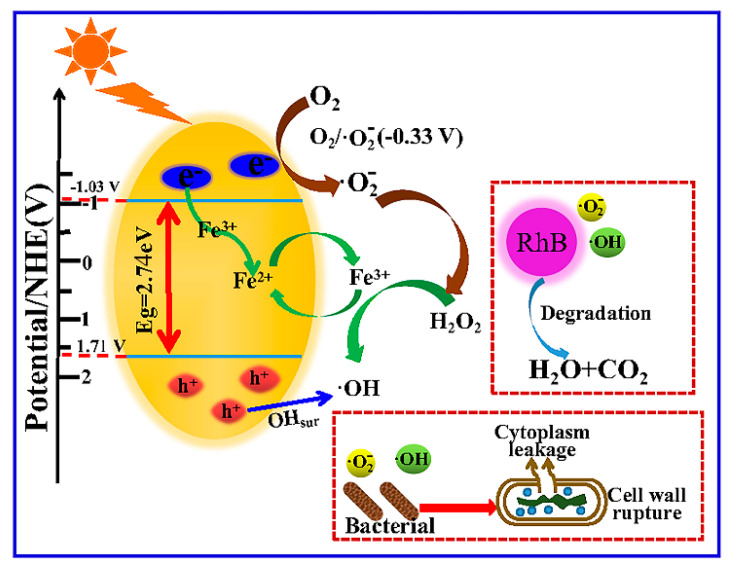
The proposed mechanism of oxygen-related radical generation in the photocatalytic system coupled with the Fenton reaction (AKCN-0.010Fe) under visible light irradiation.

Generally, reactive free radicals, including h^+^, ·OH, and $\cdot O_{2}^{-}$ are involved in photocatalytic reactions. In the present study, the main reactive free radical species for photocatalytic activities were detected by radical scavenger experiments (Figure 9). The photocatalytic activity of AKCN-0.010Fe decreased when BQ was added into the solution; therefore, $\cdot O_{2}^{-}$ was the main reactive free radical (Figure 9a). However, the degradation rate decreased slightly after adding MSDS, indicating that h^+^ is not the main radical in this photocatalytic system. IPA and TBA are the most common ·OH scavengers; however, in the current system, AKCN-0.010Fe catalyzed the decomposition of alcohol [29]. Therefore, IPA and TBA could not be used as scavengers for ·OH in this experiment. To detect the existence of ·OH radicals and their relative concentration produced by different photocatalysts, fluorescence experiments were carried out (Figure 9b). The amount of ·OH increased considerably after Fe^3+^ doping, and AKCN-0.010Fe generated the maximum amount of ·OH. The g-C_3_N_4_-based materials can catalyze the production of H_2_O_2_, and the Fenton reaction could occur when H_2_O_2_ and Fe species exist together [45]; hence, the Fenton reaction occurred under the function of AKCN-xFe. The transfer routes of photogenerated carriers during RhB degradation and sterilization by AKCN-0.010Fe are presented in Scheme 1. The photocatalytic performance of AKCN-0.010Fe can be attributed to the generation of $\cdot O_{2}^{-}$ and ·OH radicals. For n-type semiconductors, the flat band potential can be viewed as the CB potential [64]. The CB potential of AKCN-0.010Fe was determined to be −1.03 V by Equation (8).

(8) NHE=E+E(Ag/AgCl)

E is the measured potential, and E(Ag/AgCl) is the reference electrode potential (0.196 V). The CB potential of AKCN-0.010Fe (−1.03 V vs. NHE) is more cathodic than the standard redox potential of O_2_/$\cdot O_{2}^{-}$ (−0.33 V vs. NHE) [65]. Thus, the photogenerated electrons of AKCN-0.010Fe exhibit a strong reduction property and reduce O_2_ into $\cdot O_{2}^{-}$. However, the VB potential of AKCN-0.010Fe (+1.72 V vs. NHE) is more negative than the standard redox potential of OHˉ/·OH (+1.99 V vs. NHE) and H_2_O/·OH (+2.37 V vs. NHE); hence, the weak oxidation potential of photoinduced h^+^ is not sufficiently positive to oxidize OHˉ or H_2_O into ·OH radicals [66]. Furthermore, the $\cdot O_{2}^{-}$ acts as a crucial active intermediate in the generation of H_2_O_2_ (Equation (9)) [67].

(9) $$\cdot O_{2}^{-}+e^{-}+2H^{+}\to H_{2}O_{2}$$

The resulting H_2_O_2_ reacts with Fe^3+^ ions to form ·OH radicals. Since the hydroxyl groups grafted on the photocatalysts are converted into ·OH, an increase in ·OH in the dark was observed (Figure 9b). In the dark, there are more ·OH formed by AKCN and AKCN-xFe than CN, especially AKCN-0.010Fe, indicating that Fe^3+^ doping is conducive to hydroxyl groups grafting, which is consistent with the result of FTIR. Finally, $\cdot O_{2}^{-}$ and ·OH destroyed the organic macromolecules and broke the bacterial cell walls and membranes, leading to the complete conversion of organic macromolecules into nontoxic and harmless CO_2_ and H_2_O [68,69]. Therefore, it can be inferred that $\;\cdot O_{2}^{-}\;$ and ·OH have a primary role in improving the photocatalytic pollutant degradation and antibacterial performance of AKCN-0.010Fe.

## 4. Conclusions

In the present study, CN, AKCN, and AKCN-xFe were synthesized by a one-step thermal condensation method, and the AKCN-xFe sample coupled the photocatalytic reaction with the Fenton reaction, which resulted in the generation of ·OH that would not otherwise be produced in the pure g-C_3_N_4_ catalytic system. The structure and composition of the synthesized g-C_3_N_4_-based materials were characterized by SEM, TEM, AFM, XRD, XPS, EDS, FTIR, UV–visible absorption spectra, and electrochemical characterization. The AKCN-0.010Fe sample shows remarkable photocatalytic activities in the degradation of RhB and killing of *P. aeruginosa, E. coli* and *S. aureus* under visible light. The free radical trapping experiment demonstrated that $\cdot O_{2}^{-}$ and ·OH play primary roles in enhancing the photocatalytic property. In addition, an applicable photocatalytic degradation and antibacterial mechanism was proposed.

## Supplementary Materials

The following are available online at https://www.mdpi.com/2079-4991/10/9/1751/s1, Figure S1: AFM images of (a) CN, (b) ACNK, (c) AKCN-0.006Fe, (d) AKCN-0.010Fe, and (e) AKCN-0.014Fe, Figure S2: TEM images of (a) CN, (b) ACNK, (c) AKCN-0.006Fe, (d) AKCN-0.010Fe, and (e) AKCN-0.014Fe, Figure S3: EDS results of (a) CN, (b) ACNK, (c) AKCN-0.006Fe, (d) AKCN-0.010Fe, and (e) AKCN-0.014Fe, Figure S4: C 1s and N 1s XPS spectra of CN, AKCN and AKCN-0.010Fe, Figure S5: (a) Photocurrent and (b) EIS Nyquist plots of the prepared samples, Table S1: The elemental composition of all samples.
