# Supplementary material for: Photocatalytic Degradation and Antibacterial Properties of Fe3+-Doped Alkalized Carbon Nitride
Source: Nanomaterials (Basel). 2020 Sep 4;10(9):1751. doi: 10.3390/nano10091751 (PMC7558592; doi:10.3390/nano10091751)
Supplement: Supplementary file 1 [file nanomaterials-10-01751-s001.pdf]

nanomaterials

Photocatalytic Degradation and Antibacterial Properties of Fe^{3+}-Doped Alkalized Carbon Nitride

Ying Gao [1,2,3,4], Jizhou Duan [1,3,4,*], Xiaofan Zhai [1,3,4,*], Fang Guan [1,3,4], Xiutong Wang [1,3,4], Jie Zhang [1,3,4] and Baorong Hou [1,3,4]

1 Key Laboratory of Marine Environmental Corrosion and Bio-fouling, Institute of Oceanology, Chinese Academy of Sciences, Qingdao 266071, China; yinggaogy@163.com (Y.G.); guanfang@qdio.ac.cn (F.G.); wangxiutong@qdio.ac.cn (X.W.); zhangjie@qdio.ac.cn (J.Z.); brhou@qdio.ac.cn (B.H.)
2 University of Chinese Academy of Sciences, Beijing 100049, China
3 Open Studio for Marine Corrosion and Protection, Pilot National Laboratory for Marine Science and Technology (Qingdao), Qingdao 266071, China
4 Center for Ocean Mega-Science, Chinese Academy of Sciences, Qingdao 266071, China
* Correspondence: duanjz@qdio.ac.cn (J.D.); zhaixf@qdio.ac.cn (X.Z.)

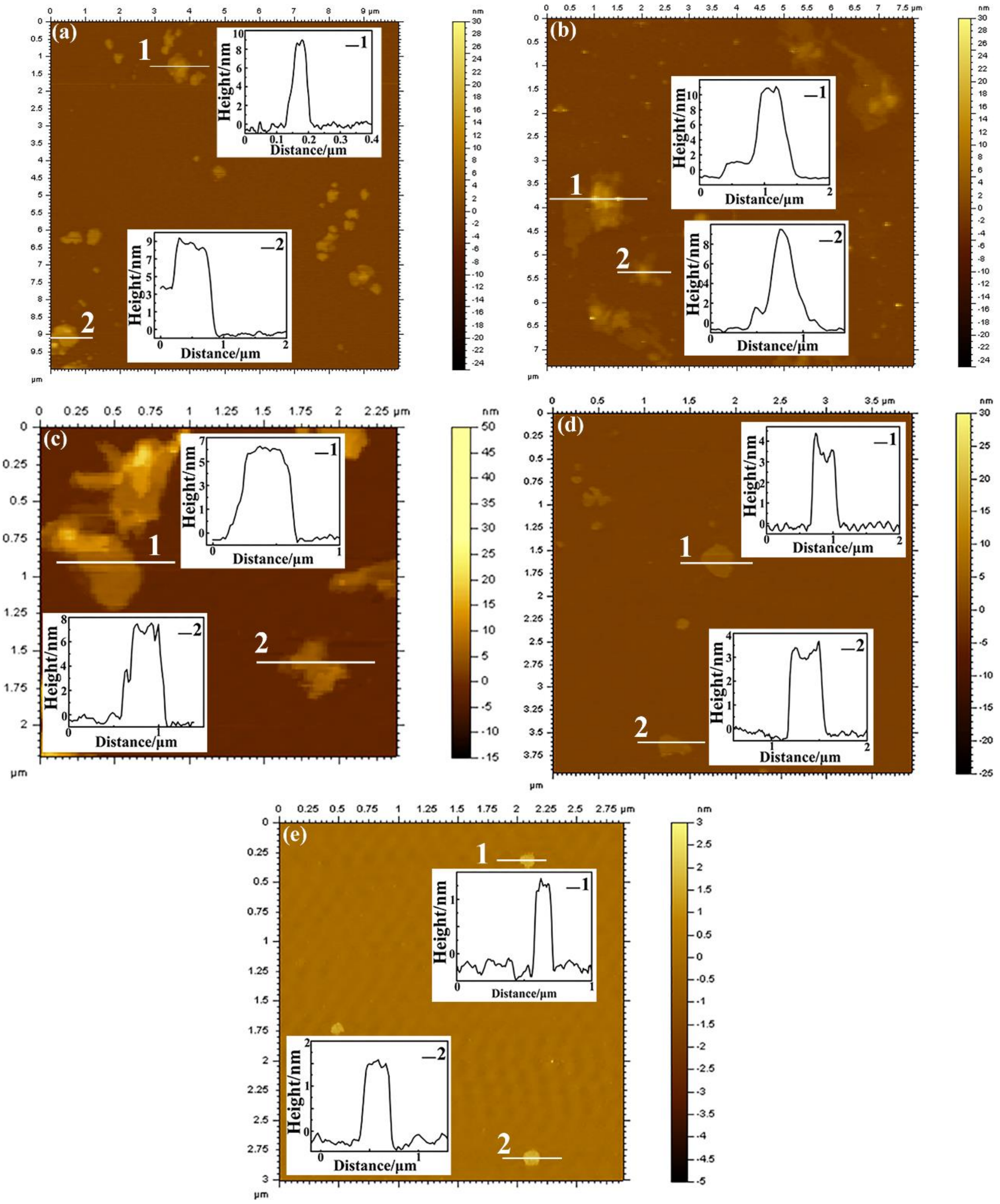

Figure S1. AFM images of (**a**) CN, (**b**) ACNK, (**c**) AKCN-0.006Fe, (**d**) AKCN-0.010Fe, and (**e**) AKCN-0.014Fe.

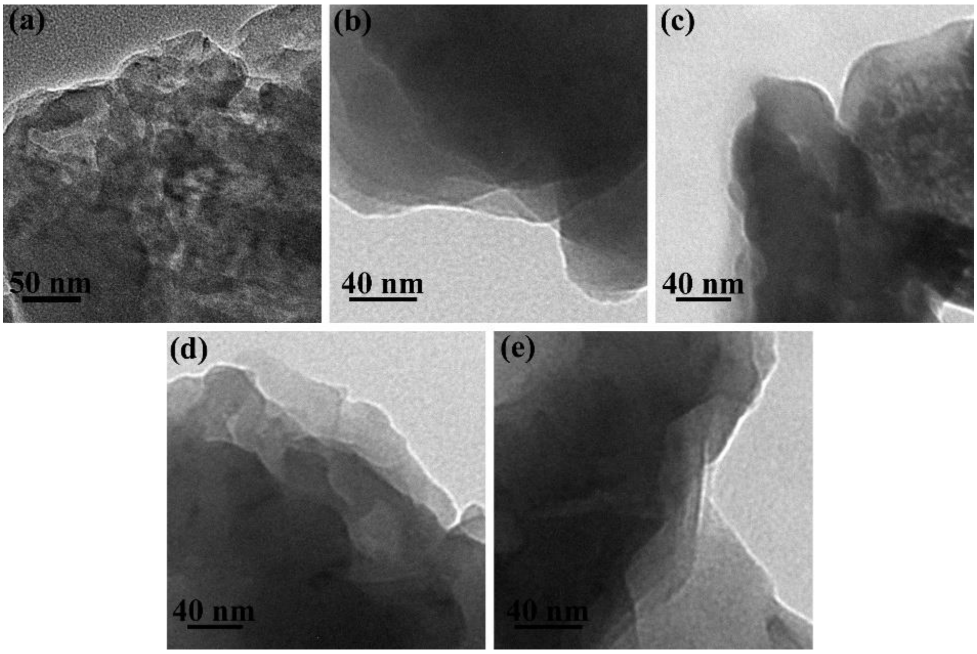

Figure S2. TEM images of (**a**) CN, (**b**) ACNK, (**c**) AKCN-0.006Fe, (**d**) AKCN-0.010Fe, and (**e**) AKCN-0.014Fe.

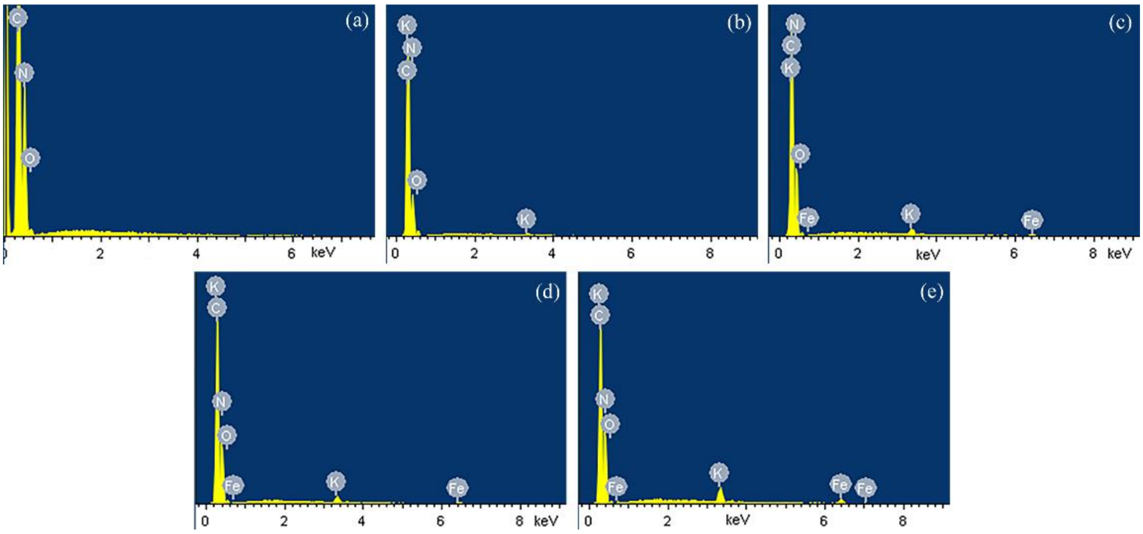

Figure S3. EDS results of (**a**) CN, (**b**) ACNK, (**c**) AKCN-0.006Fe, (**d**) AKCN-0.010Fe, and (**e**) AKCN-0.014Fe.

Table S1. The elemental composition of all samples.

Samples	Elements content (at%)				
	C	N	O	K	Fe
CN	10.40	80.56	9.05	0	0
AKCN	14.47	70.73	14.52	0.28	0

AKCN-0.006Fe	11.79	81.12	6.17	0.63	0.29
AKCN-0.010Fe	11.72	78.35	8.96	0.63	0.34
AKCN-0.014Fe	9.92	83.23	4.66	1.34	0.86

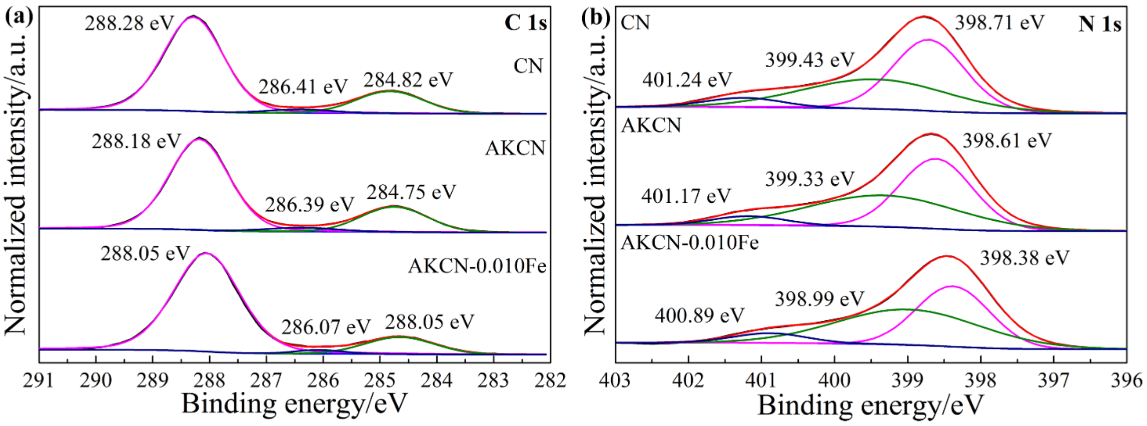

Figure S4. C 1s and N 1s XPS spectra of CN, AKCN and AKCN-0.010Fe.

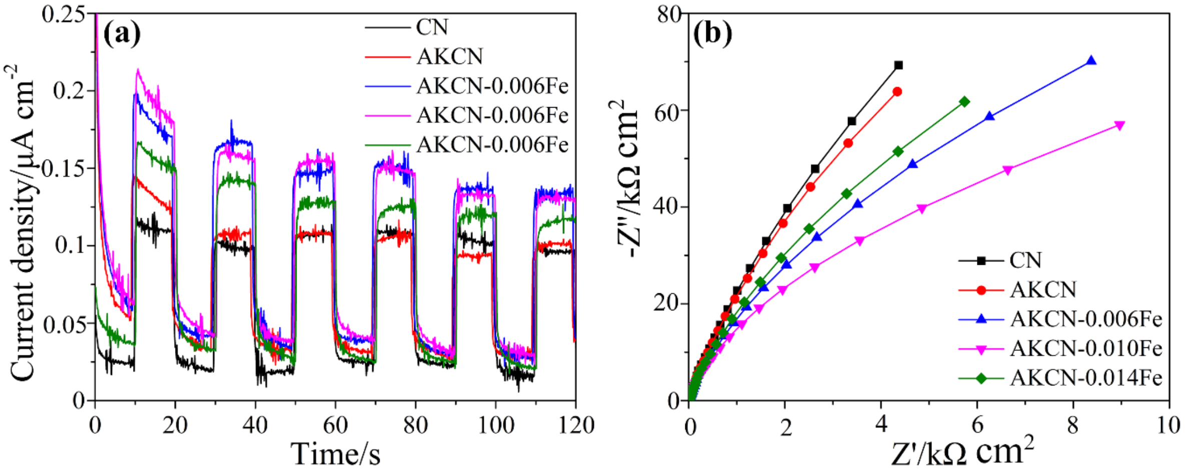

Figure S5. (a) Photocurrent and (b) EIS Nyquist plots of the prepared samples.
